# Temporal complexity in missed doses of rifampicin-sensitive anti-tuberculosis treatment: a prospective cohort study in Tanzania

**DOI:** 10.1136/bmjresp-2024-003088

**Published:** 2025-07-31

**Authors:** Lilian Tuwabunze, Kassim Salim Msaji, Alphonce Liyoyo, Proma Paul, Stellah Mpagama, Helen R Stagg

**Affiliations:** 1 Kibong’oto Infectious Diseases Hospital, Sanya Juu, Tanzania, United Republic of; 2 Department of Infectious Disease Epidemiology, London School of Hygiene & Tropical Medicine, London, UK

**Keywords:** Tuberculosis

## Abstract

**Background:**

Non-adherence to anti-tuberculosis (TB) regimens is not simplistic; rather, doses are missed in complex patterns. In a cohort of individuals being treated for rifampicin-sensitive pulmonary TB in Tanzania, we sought to examine how doses were missed across the treatment course and within a day, as well as the reasons for missed dose periods.

**Methods:**

200 participants aged ≥18 years treated with the standard 6-month regimen were recruited from March 2022 to June 2023. Missed doses were measured using evriMED pillboxes and by pill count. The reasons for up to three missed dose periods per month were collected. Patterns of missed doses—across treatment and within a day—and their reasons were visualised and described.

**Findings:**

Two participants died early in treatment, leaving 198 with missed dose data. The increase in the percentage of participants that missed any given dose as time progressed was driven by early discontinuation (median doses missed 0.0% in month 1 vs 6.7% in month 6) from treatment, as opposed to sporadic missed doses (median doses missed 3.1% in month 1 vs 4.1% in month 6). There was a median of one sporadic missed dose period (ranging between 0 and 42 doses in length) per participant. Out of all the reported reasons for missed dose periods, forgetting or forgetting and inconvenience were the most common (59.6%).

**Interpretation:**

Missing doses of anti-TB treatment is a temporally complex phenomenon and the result of the intersection of multifaceted day-to-day events in an individual’s life, with complicated implications for effective drug levels across the treatment course. This complexity limits our ability to predict an individual’s missed doses at the start of treatment.

**WHAT IS ALREADY KNOWN ON THIS TOPIC**

- Quantitative studies capturing how multifaceted day-to-day events in an individual’s life intersect with their tuberculosis (TB) diagnosis and treatment are lacking, despite some recent studies describing missed dose trajectories across the anti-TB treatment course and, occasionally, in terms of dose-by-dose missed dose patterns.

**WHAT THIS STUDY ADDS**

- In this prospective cohort study of individuals with pulmonary rifampicin-sensitive TB in Tanzania, we capture the temporal complexity of missed doses, including their accrual due to early discontinuation—but not sporadic missed doses—across the treatment course.
- We map the reasons for specific missed-dose patterns across time.

**HOW THIS STUDY MIGHT AFFECT RESEARCH, PRACTICE OR POLICY**

- The spontaneous manifestation of missed dose drivers across treatment renders accurate prediction of who will miss doses from the start of treatment difficult.
- Different missed dose patterns will have a variety of implications for drug levels in blood and disease lesions.

## Background

 Tuberculosis (TB) remains one of the most challenging infectious diseases globally, affecting about 10 million people per year.^1^ Even for drug-sensitive (DS-) TB disease, the most commonly used treatment regimen is taken over a 6-month course, with an intensive phase of four drugs for 2 months followed by a continuation phase of two drugs for 4 months.^2^ For drug-resistant (DR-) disease, treatment is even longer.^2^ These lengthy durations can lead to challenges with doses being missed. This is problematic because missed doses of anti-TB treatment are associated with prolonged infectiousness, increased transmission, higher rates of treatment failure and the development of drug resistance.^3^ Ensuring patients complete their treatment regimen is thus important for both individual health outcomes and public health.

Missed doses of anti-TB treatment are often conceptualised in simplistic and binary terms, that is, above or below a particular percentage threshold (80% or 90%). This does not, however, capture the complexity of missed dose behaviours over time.^3^ Numerous studies have indicated that missed doses increase as treatment progresses, while also fluctuating substantially during the treatment course.^3–5^ This is important because the implications of missed doses will be different during different treatment phases, for example, due to changes in bacterial load. Further, missed dose patterns are known to vary substantially from setting to setting; for example, in terms of how soon early discontinuation from treatment manifests itself.^6^

A complex array of factors leads to missed doses of anti-TB treatment. Broadly, these have been classified as economic/structural, patient (eg, comorbidities, demographic, clinical), regimen (eg, side effects), healthcare provider-patient relationship and health systems related.^7–9^ In resource-limited settings where TB burden is highest, patient-related factors, including employment, family responsibilities, social support, stigma and lack of access to healthcare facilities, can be particularly problematic.^10^ Qualitative studies also reveal that missed doses are influenced by how treatment fits into the structures of individuals’ broader lives.^11^ For example, people may have to choose between attending a clinic to replenish their medication and working to support their family.^10^

Understanding both how and why missed doses manifest is essential for designing interventions that address its root causes, as opposed to the limited support and occasional blame-based communication that patients may experience when missing doses, especially in overburdened clinical settings. This study aimed to understand the temporal dynamics of missed doses of anti-DS-TB treatment within a patient population in northern Tanzania and to provide detailed insights into the complex drivers of missed doses over time.

## Methods

### Study design and setting

This was a prospective cohort study of individuals with pulmonary DS-TB, aged ≥18 years, being treated at Kibong'oto Infectious Disease Hospital (KIDH), which is in the north of Tanzania. KIDH is located near the Mirerani mines, and thus miners are a key part of the hospital population.

The Tanzanian National Tuberculosis and Leprosy Programme (NTLP) implements Directly Observed Treatment (DOT) as the standard of care nationwide. As part of this approach, patients are encouraged to identify a treatment supporter—often a family member—who observes and documents the intake of medications, especially during the continuation phase of treatment.^12^ Despite the widespread use of this model, implementation challenges are known to occur. These include limited training and supervision of treatment supporters, inconsistent follow-up by health workers and lack of tailored support for patients with complex socioeconomic or clinical needs. In busy public sector hospitals, such as the one where this study is conducted, resource constraints further limit the availability of personalised adherence counselling or structured psychosocial interventions.

Individuals were consented and enrolled between March 2022 and June 2023, and post-treatment follow-up was completed in June 2024.

### Participants

Eligible participants had bacteriologically confirmed (through sputum sampling; namely, smear examination, culture or GeneXpert) pulmonary disease deemed sensitive to rifampicin through GeneXpert. They were starting the standard 6-month DS-TB treatment regimen: 2 months of isoniazid (75 mg), rifampicin (150 mg), ethambutol (275 mg) and pyrazinamide (400 mg) followed by 4 months of isoniazid (75 mg) and rifampicin (150 mg) (2HRZE/4HR), all dosed daily. Medications were generally issued as fixed dose combination pills (FDCs), and dosage was determined by a participant’s weight. Participants were instructed to take their TB medication once daily, as per national treatment guidelines. Participants provided informed consent in order to be enrolled in the study.

Participants who lived a long distance (>200 km) from KIDH were not recruited due to logistical constraints that would have resulted in inconsistent follow-up and data completeness for these individuals versus the rest of the cohort. The study’s missed dose monitoring required regular in-person visits and phone-based check-ins, which were not reliably feasible for individuals in remote regions due to transportation barriers and limited mobile network access.

### Missed dose (non-adherence) measurement

Non-adherence is technically defined as deviations from the agreement made between participants and healthcare providers at the start of treatment about how treatment will be taken.^13^ Within this study, we also considered as of interest doses missed due to side effects where the healthcare provider agreed with the participant that they could be missed, and thus refer to ‘missed doses’ rather than ‘non-adherence’ throughout this manuscript.

In our prospective cohort, we captured missed doses through two mechanisms: electronically (evriMED pillboxes, a type of medication event reminder monitor that is, a container for medication that digitally records when it is opened) and pill counts.

#### Electronic data capture (pillboxes)

At enrolment and subsequent monthly visits, participant’s medication was dispensed into evriMED 500 pillboxes (Wisepill Technologies) by a KIDH healthcare worker. These medication monitoring devices capture pillbox opening as a proxy measure for a dose being taken and were used in this study without the reminder mechanism (alarm, lights) being set. Each day, the devices also record a heartbeat to check that the pillbox is working. Data as to pillbox openings and heartbeats were uploaded from the pillboxes to the Wisepill cloud at monthly appointments at KIDH, before the pillbox was refilled with new medication. As the study was also collecting pharmacokinetics data for which dose-taking needed to be very reliable during the first 2 weeks of treatment, in this period a healthcare worker contacted participants daily to remind them to take their medication.

Participants were instructed to open the pillbox only at the time of dosing. They were advised not to open the device for any other reason to ensure accurate data capture.

#### Monthly pill count

Before medications for the next month were dispensed, the KIDH healthcare worker counted the number of pills remaining in the pillbox.

### Missed dose data coding

#### Electronic data capture

Pillbox data were cleaned as per online supplemental table S1 to create two variables: a ‘main’ missed dose variable and one for sensitivity analyses. Broadly for both variables, if the pillbox was opened at least once a day, this was coded as a dose taken. If the pillbox was not opened, but a heartbeat was present, this was coded as dose not taken. Pillbox data could be overwritten by other data, for example, the first dose of treatment always being taken at the clinic under observation (online supplemental table S1). The two variables differed in their treatment of (1) information reported on ‘pocket doses’ (ie, where participants took multiple days’ worth of doses out of their pillboxes on a single day; see the Reasons for missed dose patterns section) during monthly clinic visits; (2) medication due to be taken after the last visit of the study; and (3) loss to follow-up. The main missed dose variable assumed more missed doses than the sensitivity analysis variable. Only data across a 168-day period (28 days per month over 6 months) were considered.

### Reasons for missed dose patterns

Each month, KIDH healthcare workers examined the missed dose data recorded by the pillboxes. For research purposes only, they then had a discussion with the participant about what enabled them to take their medication during that month (‘When you were taking your anti-tuberculosis pills this month, what helped you to take them each day?’).

Further, the healthcare worker selected up to three missed dose periods each month and asked the question ‘When you did not take your anti-TB pills during this part of the month, what caused problems for you?’ The healthcare worker recorded the reason for the missed dose for the period, either from a list of potential items (forgot, feeling better, inconvenient, feeling unwell, unable due to other reasons (travel, out of medication), side effects or other issues with the regimen, lack of support) or in free text. The *txttool* Stata command and manual review was used to group the free text responses. The grouped free text responses and information from the list of potential items were combined to generate a final set of reasons for missed doses: pocket doses, inconvenient, forgot, unable/lack of support, side effects/unwell and felt well.

### Collection of other study data

Baseline data on demographic information and medical history (including self-reported comorbidities) were recorded. At the monthly clinic visits, additional data capture included changes in treatment regimens, comorbidities and side effects during treatment. Sputum smear and culture results were obtained from medical records at 2, 3 and 6 months.

All study data apart from the pillbox data were collected and managed using REDCap (Research Electronic Data Capture) electronic data capture tools hosted on a server at the London School of Medicine & Tropical Hygiene.^14 15^ REDCap is a secure, web-based software platform designed to support data capture for research studies, providing (1) an intuitive interface for validated data capture; (2) audit trails for tracking data manipulation and export procedures; (3) automated export procedures for seamless data downloads to common statistical packages; and (4) procedures for data integration and interoperability with external sources.

### Statistical analysis

Analyses were done using Stata V.15.1 (StataCorp, College Station, Texas, USA).

#### Missed dose patterns from pillboxes

Doses taken were described using the following summary measures: median percentage of doses taken overall, during the intensive phase and during the continuation phase and percentage of participants achieving a 100% and 80% threshold of doses taken. Doses with missing information were excluded from the denominator of these calculations.

The patterns of doses missed over time, grouped by different percentage intervals, were graphically visualised using lasagna plots created in Excel (Microsoft Corporation). Stacked area plots were used to visualise doses missed due to early cessation (discontinuation) of treatment vs doses missed sporadically (ie, suboptimal dosing implementation) using the *rarea* command in Stata. To determine the doses missed due to early discontinuation, per individual, we looked for the last timepoint at which a dose was taken before all other doses up to and including dose 168 were missed. This division of types of missed doses is in line with published taxonomies.^3 16^ The number of gaps during treatment due to sporadic missed doses and their length were described using scatter plots. Line graphs were used to visualise changes in medication timing over the treatment period.

Both the main missed dose variable and the sensitivity analysis variable were used throughout these analyses.

#### Reasons for missed doses

The reasons for missed doses per gap period were graphically summarised by months in treatment.

#### Comparison between missed dose data from pillboxes and monthly pill count

The percentage of doses taken over the treatment period recorded by the monthly pill counts and pillboxes (main missed dose variable and sensitivity analysis variable) was compared using box plots/violin plots using *vioplot* command in Stata. Percent agreement as to whether individual participants were recorded as having no missed doses (ie, 100% doses taken) between the two measures was calculated.

#### Patterns in timings for dose taken

To examine when within a day a dose of medication was taken, a 24-hour window of 04:00 to 03:59 was set. When multiple openings were recorded within a day, we assumed the first to be the time that the dose was taken. Medication taken in the morning was defined as a dose taken between 04:00 and 14:00. Medication taken after 14:00 to the end of the 24-hour window was considered an evening dose.

### Patient and public involvement

Patients were involved in the development of the research questions, but not the study design, recruitment, outcome measures, dissemination plans and conduct of the study. The study findings will be disseminated through open access scientific publications, stakeholder networks and national/international meetings.

## Results

### Participants

Of the 695 people with presumed TB at KIDH during the time window, 200 (28.8%) met the inclusion criteria (online supplemental figure S1). The majority of enrolled participants were male (185/200, 92.5%), and the mean age was 41.6 years (table 1). Eighty-three and a half per cent (167) had limited education, having only completed primary school or lower. Fifty-two participants (26.0%) reported having a comorbid condition, such as diabetes, HIV/AIDS and other lung disease, especially pneumoconiosis. Having a history of TB was reported by 25.5% (51/200) of the participants. Forty-four per cent (88/200) of participants were underweight, potentially suffering from malnutrition. All the participants were on 2HRZE/4HR, dosed with FDCs, at the start of treatment.

**Table 1:** Baseline characteristics of cohort (N=200)

	N (%)
Age, in years	
Mean age (SD)	41.6 (11.2)
18–29	33 (16.5%)
30–39	43 (21.5%)
40–49	79 (39.5%)
≥50	45 (22.5%)
Sex	
Female	15 (7.5%)
Male	185 (92.5%)
Education	
No education	29 (14.5%)
Some primary school	21 (10.5%)
Primary school completed	117 (58.5%)
Any secondary school	28 (14.0%)
University	5 (2.5%)
Smoking history	
Current smoker	14 (7.0%)
Former smoker	95 (47.5%)
Never smoked	90 (45.0%)
Missing	1 (0.5%)
Alcohol consumption	
Advised to reduce	67 (33.5%)
Not advised to reduce	133 (66.5%)
Recreational drug use	
Yes	4 (2.0%)
No	196 (98.0%)
GeneXpert results	
TB positive, rifampicin resistance not detected	199 (99.5%)
TB positive, rifampicin resistant indeterminate	1 (0.5%)
Sputum smear result	
Negative or scanty†	119 (65.5%)
1+	35 (17.5%)
2+	22 (11.0%)
3+ or more	11 (5.5%)
Missing	1 (0.5%)
Culture results	
Negative	41 (20.5%)
Positive	153 (76.5%)
Test failed	3 (1.5%)
Test not done	1 (0.5%)
Missing	2 (1.0%)
Extrapulmonary disease in addition to pulmonary	
Yes	8 (4.0%)
No	192 (96.0%)
Previous TB	
Yes	57 (28.5%)
No	143 (71.5%)
Missing	1 (0.5%)
Intended adherence support	
None	5 (2.5%)
DOT-clinic	2 (1.0%)
DOT-community	3 (1.5%)
DOT-family member	190 (95.0%)
Body mass index	
Overweight (≥25)	11 (5.5%)
Normal (≥18.5–<25)	101 (50.5%)
Underweight (<18.5)	88 (44.0%)
HIV/AIDS*	
HIV, taking medication	5 (2.5%)
HIV, not taking medication	4 (2.0%)
No HIV reported	191 (95.5%)
Diabetes*	
Diabetes, taking medication	6 (3.0%)
Diabetes, not taking medication	2 (1.0%)
No diabetes	192 (96.0%)
Other lung disease*	
Other lung disease, taking medication	15 (7.5%)
Other lung disease, not taking medication	21 (10.5%)
No other lung disease	164 (82.0%)

* Other comorbidities, including hepatitis B/C, malaria, sickle cell anaemia and mental health conditions were not reported at baseline among the 200 participants.

† Twelve scanty smear results.

DOT, Direct Observation Therapy; TB, tuberculosis.

### Patterns of missed doses

Of the 200 participants, 2 (1.0%) died from TB within the first month of treatment and their evriMED pillboxes were not recovered. Of the remaining 198 participants, 34.3% (68/198) took all 168 doses during the treatment period and 10.6% (21/198) took less than 80% of their doses using the main missed dose variable. The median percentage of doses taken was 99.4% (IQR 97.0–100%). A lower percentage of doses was taken during the intensive phase (median=96.5%; IQR 96.5–96.5%) compared with the continuation phase (median=99.1%, IQR 96.4–100%).

**Figure 1:**
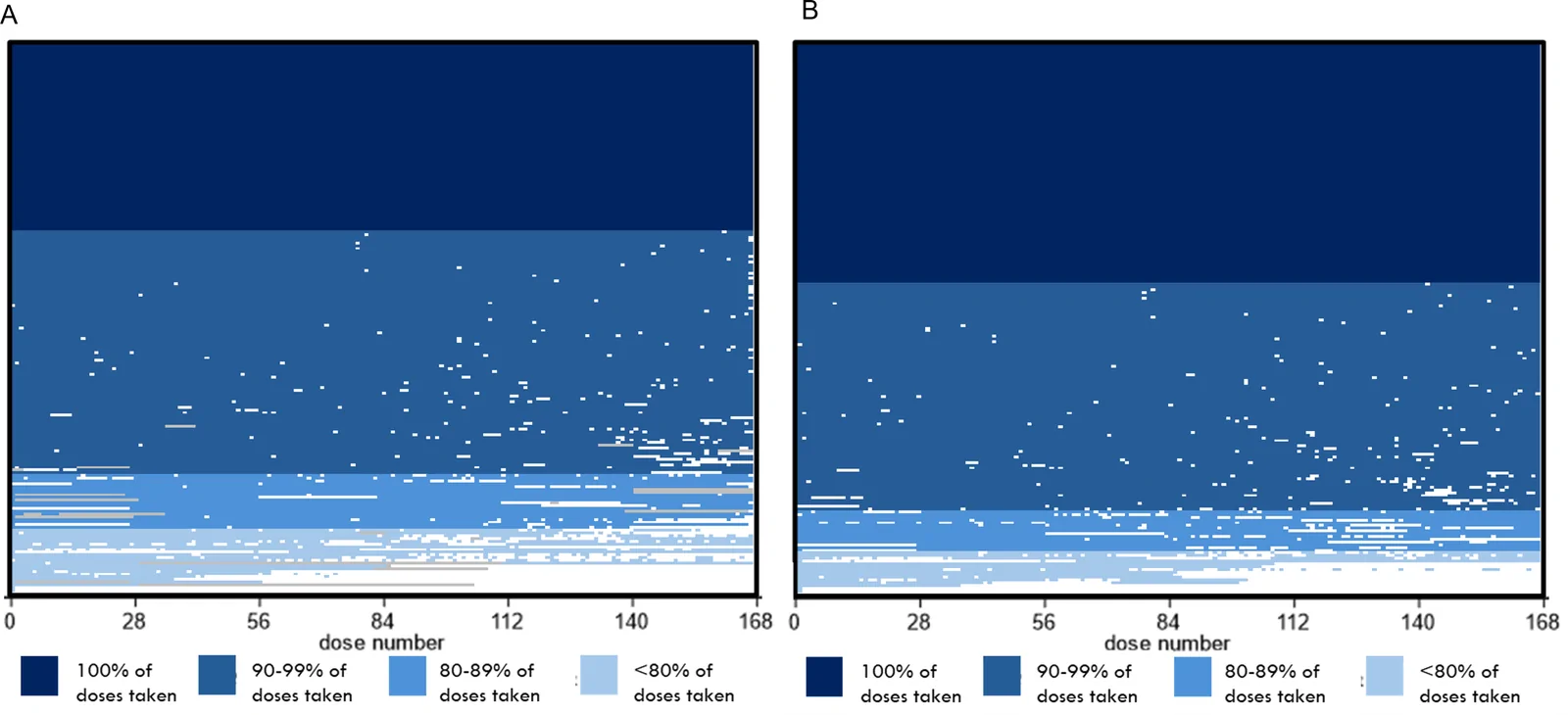
Lasagna plots of doses taken (N=200). Lasagna plots for the main missed dose variable (A) and the sensitivity analysis missed dose variable (B). Each row in the figure represents a participant, ordered by percentage of doses taken. This was calculated as a percentage of 168 doses and then grouped: <80%, 80–89%, 90–99% and 100%. Rows are coloured by dose taken groups, as per the legend. White indicates doses not taken. Grey indicates when pillbox data were missing or unreliable (eg, box failure or lost box).

Lasagna plots were used to examine missed dose patterns (figure 1). Short periods of sporadic doses were common throughout the treatment period, regardless of a participant’s overall missed dose level (figure 1A).

Within the sensitivity analysis, the percentage of participants taking all of their doses and 80% of their doses increased to 41.4% (vs 34.3% before) and 90.4% (vs 89.4% before), respectively (figure 1B).

**Figure 2:**
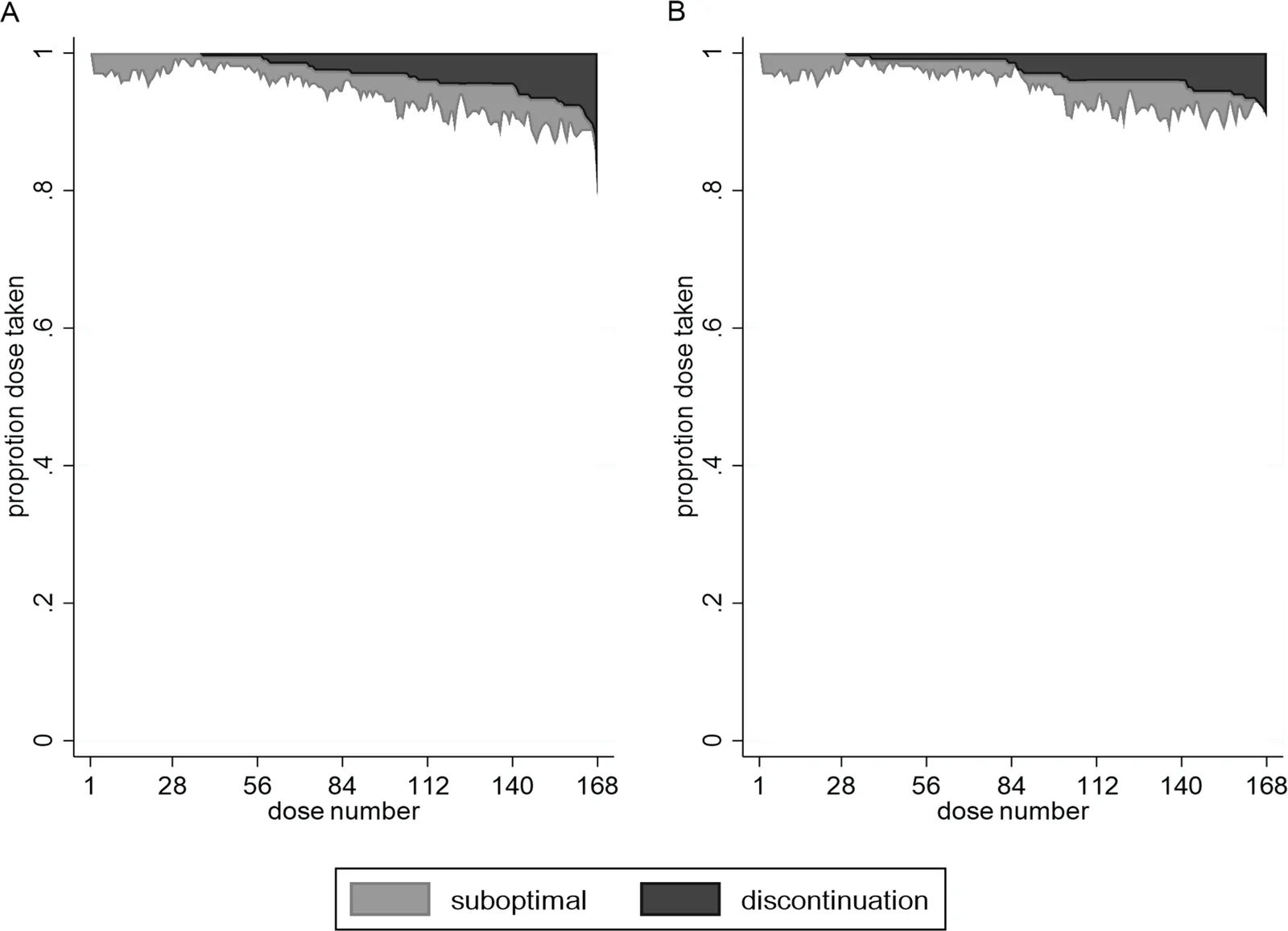
Relative contribution of early discontinuation versus sporadically missed doses over time using (A) the main missed doses variable and (B) the sensitivity analysis missed dose variable (N=198). Sporadic missed doses (light grey), early discontinuation (dark grey).

Next, we examined the relative importance of sporadic missed doses (eg, suboptimal dosing implementation) versus early discontinuation as types of missed doses within the cohort (figure 2). In the first month of treatment, sporadic missed doses were the primary reason for missed doses. As treatment progressed, there was a gradual increase in the proportion of participants missing doses, largely due to an accrual of individuals undergoing early discontinuation. Across month 6, a median of 11.3% of doses were missed, of which 62.5% were missed due to early discontinuation and 37.5% were sporadically missed. Ten per cent of participants (20/198) discontinued early without also sporadically missing doses. In the sensitivity analysis, this was fewer than 5% (9/198) of the cohort.

**Figure 3:**
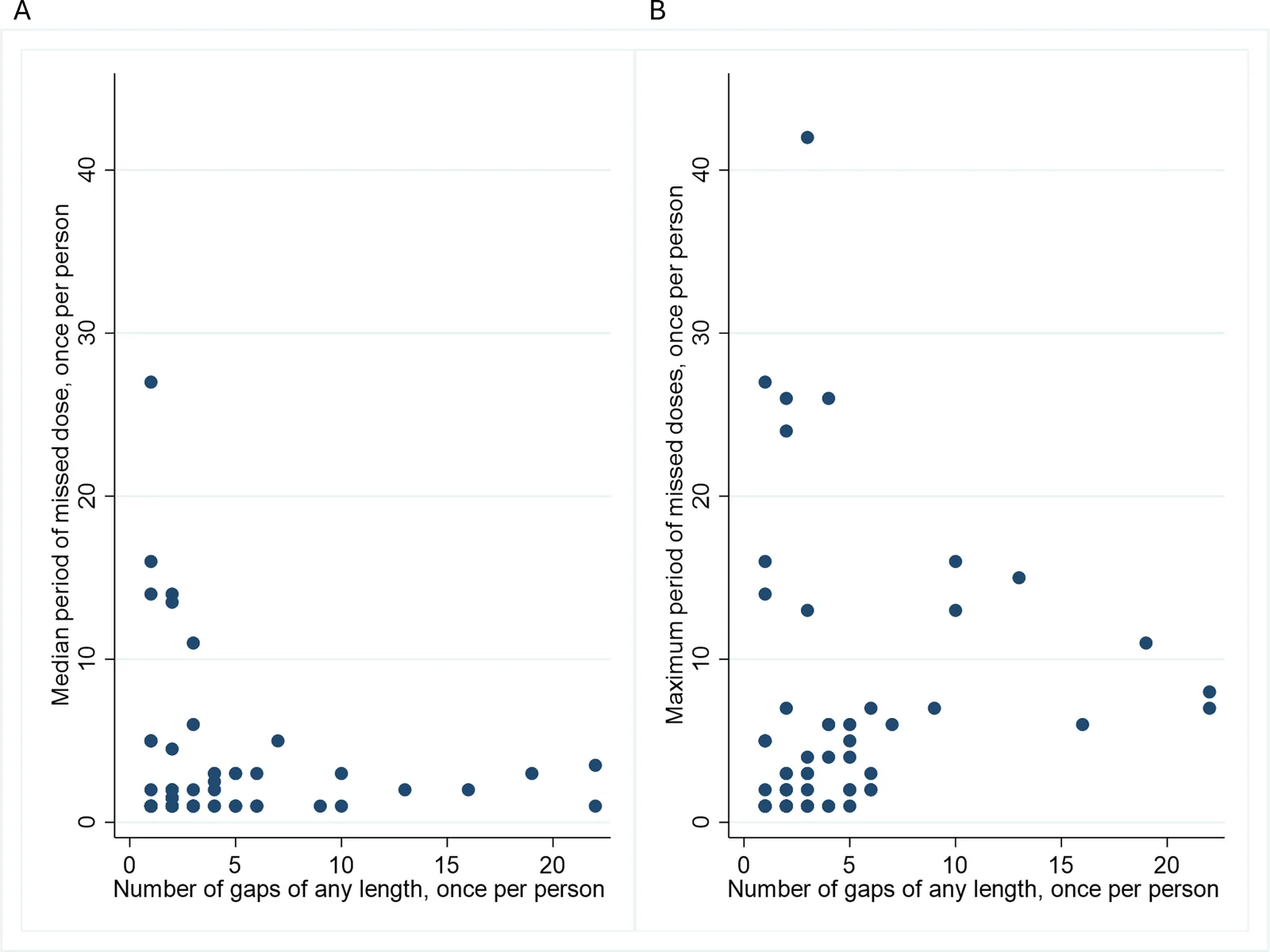
Missed dose gaps among those with sporadic missed doses (N=110). Scatter plot of the median (A) and maximum (B) length of gaps for an individual against the number of gaps of any length for an individual, using the main missed dose variable.

Specifically examining sporadic missed doses (110 individuals, online supplemental figure S2), 406 missed dose gaps were observed. Among individuals with fewer than four gaps during their treatment, the median number of doses missed across their gaps ranged from 1 to 27, with a maximum ranging from 1 to 42 (figure 3A,B). In comparison, among individuals with four or more gaps, the median ranged from one to five, with a maximum ranging from 1 to 16 (figure 3A,B). Using the sensitivity analysis missed dose variable, there were 385 gaps, with similar ranges of median and maximum lengths across an individual (online supplemental figure S2).

### Timings of doses taken

The majority of participants (180/198) changed the timing of their dose (ie, from morning to the evening) at least once during their treatment period. Even among participants who did not miss any doses of their treatment, only 21.3% (17/80) took medications at a consistent time each day (online supplemental figure S4A). Among individuals who missed <10% of their doses, timing fluctuations were observed, with more variability later in treatment (online supplemental figure S4B) or irregular dosing times throughout the whole treatment period (online supplemental figure S4C). As the proportion of missed doses increased, the dose timing became more variable, suggesting irregular dosing times with little consistency (online supplemental figure S4C–E). We also observed multiple box openings in a day outside of clinic visit days and irrespective of the overall proportion of missed doses (online supplemental figure S4).

### Comparison between electronic monitoring and pill counts

A higher percentage of participants took all 168 doses of their treatment according to the pill counts (125/198, 63.1%) versus the pillbox (68/198, 34.3%) (online supplemental figure S3). Moderate agreement was observed between the pill count and main missed dose variable from the pillbox (68.7% agreement, kappa=0.46) and between the pill count and the sensitivity analysis missed dose variable (68.2% agreement, kappa=0.39). The agreement was better between pill count and main missed dose variable from the pillbox at an 80% threshold of doses taken (93.9% agreement, kappa=0.60) and between the pill count and the sensitivity analysis missed dose variable (92.9% agreement, kappa=0.50).

### Temporal variability in factors leading to missed doses

**Figure 4:**
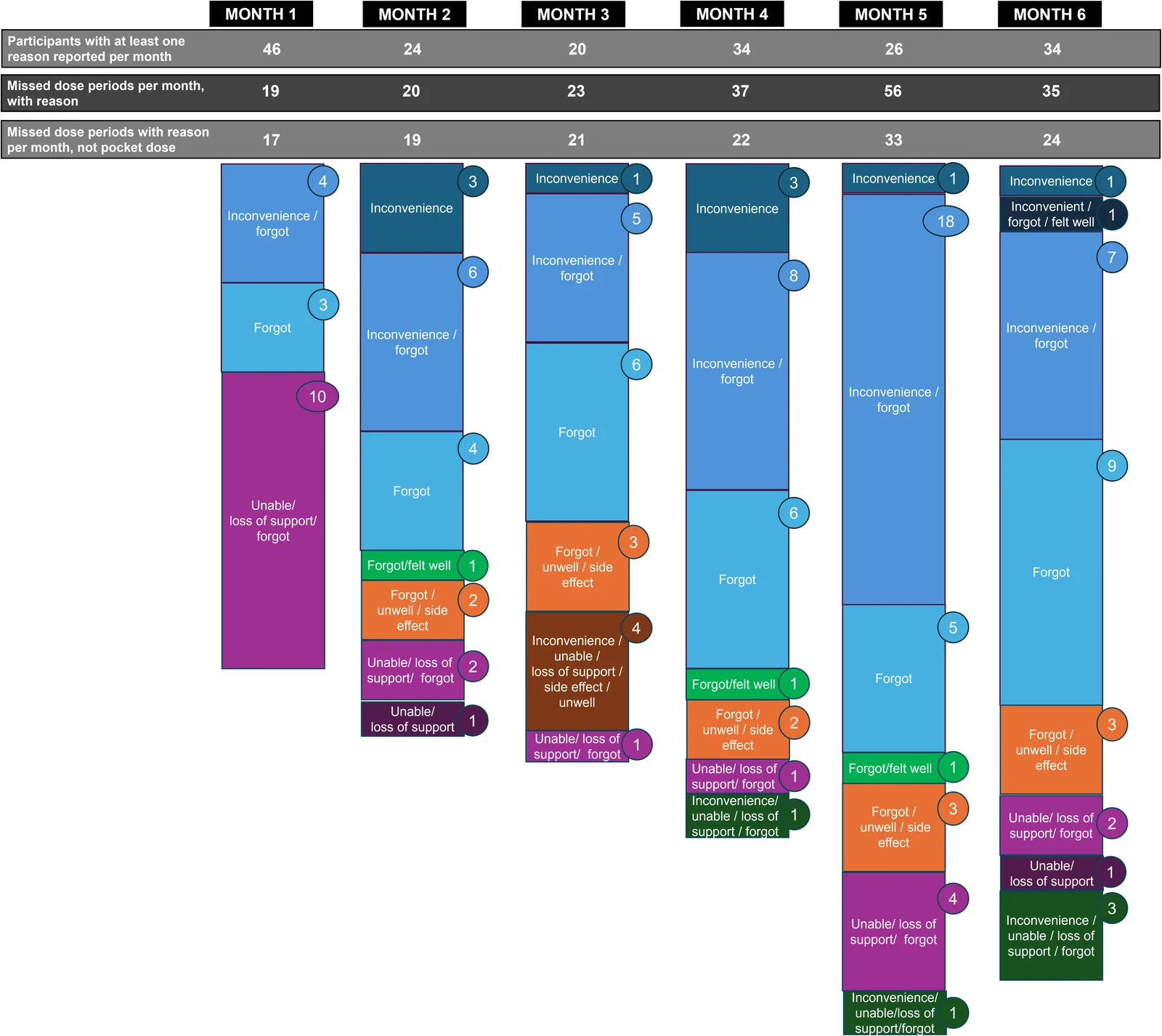
Reasons for missed doses across the treatment period. The reasons for periods of missed doses (sporadic or early discontinuation) were reported for up to three missed dose periods per month by the participant. Numbers in circles indicate the number of periods that occurred for that reason (or group of reasons) within a month. Multiple missed dose periods can be attributed to a participant in a particular month. If the missed dose period spanned more than 1 month, it was reported across both months. Main missed dose variable used; missed dose periods explained by pocket doses not included within the figure. A total of 106 participants reported at least one reason for missed doses across the treatment period.

The reasons reported by participants for missed dose periods are charted in figure 4 and also in lasagna plots (online supplemental figure S5). One hundred and six participants reported a reason for a missed dose period over the study period; this changed by month, with the highest number of participants reporting reasons in the first, fourth and last month of treatment (figure 4). Across time, the combined reason of inconvenience/forgot as well as forgetting alone was common among the pool of reasons (ranging between 41.2% and 69.7% of reasons given per month). Unable/lack of support/forgot as a combined reason was most dominant in month 1 (58.8%). Further details provided by participants indicated that family responsibilities (including taking care of sick relatives), attending funerals, travel and homelessness underlay the inconvenience of dose-taking/being unable to take doses. Feeling well (always reported in combination with other factors) was less commonly present within the pool of reasons for missed dose periods than feeling unwell/side effects.

Median gap length for each major grouping of reasons did not vary substantially by reason (online supplemental table S2), but wider ranges were associated with forgetting to take a dose, inconvenience and being unable/a lack of support.

## Discussion

In this study of missed doses of anti-TB treatment in a cohort of individuals in the north of Tanzania, we observed extensive variability over time, both in terms of the patterns of missed doses and the reasons for these patterns. Most participants experienced brief, infrequent lapses (sporadic missed doses) across the treatment course and a subset ceased treatment early (early discontinuation), predominantly during the continuation phase. It was the latter that led to increases in the proportion of participants that missed any given dose of treatment as time progressed. Switches between morning and evening dosing of medication were observed, as well as more erratic time changes within a day. Where reasons for missed dose patterns were given, inconvenience and forgetting were common; these were also two of the reasons associated with the longest missed dose periods.

To date, few studies have examined missed dose patterns dose-by-dose for anti-TB treatment. In a previous study using data from the control arm of an intervention trial to improve adherence in China, patterns were also found to be complex, with missed doses accruing over time.^17^ The study highlighted the importance of temporal events (weekends, holidays, the transition between the initiation and continuation phases of treatment) as factors influencing missed doses and, similarly to our work, found a preponderance of short sporadic missed dose gaps. In a retrospective study among individuals with multidrug-resistant TB in the Philippines, the median length of a sporadic missed dose period was 1.4 days (ranging from 1 to 37)—similar to the DS-TB population in this study—and the importance of the length of sporadic missed dose periods on the risk of poor treatment outcomes was highlighted.^18^

Although the literature is rich in quantitative and qualitative studies examining risk factors for missed doses, in this study, we have additionally sought to map reasons for missed doses to specific missed dose patterns. Examining individual’s data in detail, we found disruptions to daily routines from taking medication and clashes with work commitments to derail dose-taking, and sometimes the use of ‘pocket doses’ as a workaround. This fits with preceding work on the difficulties that individuals have integrating their TB diagnosis and treatment into their day-to-day lives.^9 19^ These findings align with the wider literature, where stigma, psychological distress and socioeconomic barriers have been identified as major obstacles to dose-taking. Stigma, as highlighted by Nabaziwa *et al*, deters patients from seeking treatment and integrating medication into their routines,^20^ while Chen *et al* emphasised the importance of societal support for dose-taking.^21^ Psychological distress, such as anxiety and depression, correlates with missed doses,^22 23^ and socioeconomic challenges like transportation costs further complicate consistent treatment.^24 25^ Throughout, it is clear that the factors influencing dose-taking dynamically fluctuate over time, posing challenges to patients and healthcare providers alike. Our dataset is also unique in examining individuals altering the timings of their doses within a day.

The strengths of our study include the use of two dose-taking measures (pillboxes and pill counts), one of which provided dose-by-dose data. Importantly, we were able to obtain information on ‘pocket doses’ (and from other data sources) to adjust the data from the pillboxes, where necessary. Further, the use of the pillboxes allowed us to examine changes in dose timings during a day, and we were able to collect data on the reasons driving different missed dose patterns. Our cohort includes individuals with a series of comorbidities (such as diabetes, malnutrition, HIV and silicosis). Vice versa, our exclusion of individuals who lived far from the clinic placed limits on generalisability, given their potential to have lower dose-taking. Selection bias arising from individuals not consenting to the study may have led to an underestimation of how common it is to miss doses, if those individuals were more likely to do so. We may have influenced adherence patterns due to (1) the daily reminders to take medication during the first 2 weeks of treatment and (2) the monthly discussions with participants about their adherence patterns.^26 27^ Data captured from the pillboxes likely reflect both adherence behaviours and engagement with the device.^28^ We captured data on reasons for missed dose periods for up to three periods a month; capturing data for all missed dose periods would have provided a more complete dataset.

Further work examining dose-by-dose missed dose patterns in different settings, the reasons that drive them and their impact on blood and lesion drug levels would be beneficial. Scientific advances to generate a gold standard tool for adherence monitoring would be hugely helpful in both research and clinical terms. How non-adherence manifests (including dose-by-dose) in normal patient populations towards the modern anti-TB regimens such as the rifapentine-moxifloxacin DS-TB regimen^29^ is unknown; this contributed to the conditional recommendation of this regimen by the WHO.^2^ Without these pieces of the puzzle, it is difficult to design effective interventions to boost dose-taking, which will likely require elements of tailoring towards individual patients. Within the context of this setting, the widespread use of family members as DOT supporters reflected the national standard of care under NTLP guidelines. While this model offers practical advantages in terms of accessibility and social support, it may also introduce variability in DOT quality, depending on the training, engagement and resources available to family supporters. There may be an opportunity to strengthen this model by providing structured education, supportive supervision and potentially integrating digital adherence tools to assist treatment supporters. Evidence on alternative, digital, interventions that improve adherence and treatment outcomes in low-income settings is currently minimal.^30^

Missing doses of anti-TB treatment is temporally complex and not easily summarised across an individual’s treatment course. As well as arising from the difficulties that patients have fitting their TB diagnosis and treatment into their day-to-day lives, missing doses is driven by life events that arise spontaneously and fluctuate across the treatment course. This makes predicting at baseline which individuals will experience issues adhering to their treatment inherently difficult. Variability in how missed doses manifest and their drivers further emphasises the need for targeted interventions to improve patient outcomes.

## Supplementary material

10.1136/bmjresp-2024-003088 online supplemental file 1

10.1136/bmjresp-2024-003088 online supplemental file 2

## Data Availability

Data are available upon reasonable request.
