# Supplementary material for: Temporal complexity in missed doses of rifampicin-sensitive anti-tuberculosis treatment: a prospective cohort study in Tanzania
Source: BMJ Open Respir Res. 2025 Jul 31;12(1):e003088. doi: 10.1136/bmjresp-2024-003088 (PMC12315038; doi:10.1136/bmjresp-2024-003088)
Supplement: online supplemental file 2 [file bmjresp-12-1-s002.docx]

**Supplemental Material**

Temporal complexity in missed doses of rifampicin-sensitive anti-tuberculosis treatment: a prospective cohort study in Tanzania

**AUTHORS**

Lilian Tuwabunze^1^*, Kassim Salim Msaji^1^*, Alphonce Liyoyo^1*^, Proma Paul^3*#^, Stella G Mpagama^1^^, Helen R Stagg^3^^

*co-first authors

^co-senior authors

**Affiliations**

1. Kibong'oto Infectious Disease Hospital, Siha, Tanzania

2. University of Liverpool, Liverpool, UK

3. London School of Hygiene & Tropical Medicine, London, UK

**Keywords**

tuberculosis; drug-sensitive; non-adherence; Tanzania

Table of Contents

[Table S1. Coding of missed dose data 3](#_Toc200032016)

[Table S2. Missed dose period length for a type of reason 3](#_Toc200032017)

[Figure S1. Flow chart of study population 5](#_Toc200032018)

[Figure S2. Missed dose gaps among those with sporadic missed doses (N=110). 6](#_Toc200032019)

[Figure S3. Differences in pillbox data and monthly pill counts 7](#_Toc200032020)

[Figure S4. Patterns in timings of doses taken, for selected participants. 8](#_Toc200032021)

## Table S1. Coding of missed dose data

Coding of the pillbox data and sources of additional data. Two variables were used within the paper- the main missed dose variable and the variable used for the sensitivity analyses. Bold highlights the differences between the two measures. *Where the date for the pocket doses could be confirmed. ^Coded as pillbox failure/lost pillbox in lasagna plot.

| **Recorded information** | **Main missed dose variable** | **Sensitivity analysis missed dose variable** |
| --- | --- | --- |
| Pillbox opened (coded as such if pillbox opened, heartbeat may or may not have been present, and these data were not later over-written by other data sources) | Dose taken | Dose taken |
| Pillbox not opened (coded as such if pillbox not opened, heartbeat was present, and these data were not later over-written by other data sources) | Dose missed | Dose missed |
| Pillbox failure (coded as such if pillbox not opened, heartbeat was not present, and these data were not later over-written by other data sources)^ | Missing data | Missing data |
| Pillbox lost^ | Missing data | Missing data |
| Dose taken at clinic under observation (could over-write the pillbox data) | Dose taken | Dose taken |
| Medication believed to be self-administered at the end of treatment due to last visit being prior to the end of the treatment period (could over-write the pillbox data) | **Dose missed** | **Dose taken** |
| Lost to follow-up (could over-write the pillbox data) | **Dose missed from lost to follow-up date, which was the last time the clinic saw the participant** | **Dose missed from lost to follow-up date plus 28 days i.e. assumes that the participant took the medication issued to them during the visit when the clinic last saw them** |
| Death and treatment failure (could over-write the pillbox data) | Dose missed from death/treatment failure date | Dose missed from death/treatment failure date |
| Pocket dose* (could over-write the pillbox data) | **Dose missed** | **Dose taken** |

## Table S2. Missed dose period length for a type of reason

| **Reason** | **Missed dose period length (doses)** | | |
| --- | --- | --- | --- |
|  | **Median** | **IQR** | **Range** |
| Forgot | 1 | 1-2 | 1-24 |
| Felt well | 2 | 1-4 | 1-5 |
| Inconvenient | 1 | 1-2 | 1-15 |
| Side effects/unwell | 1 | 1-3 | 1-5 |
| Unable/lack of support | 2 | 1-4 | 1-24 |

##

## Figure S1. Flow chart of study population

All eligible individuals were invited to participate in the study. TB tuberculosis.


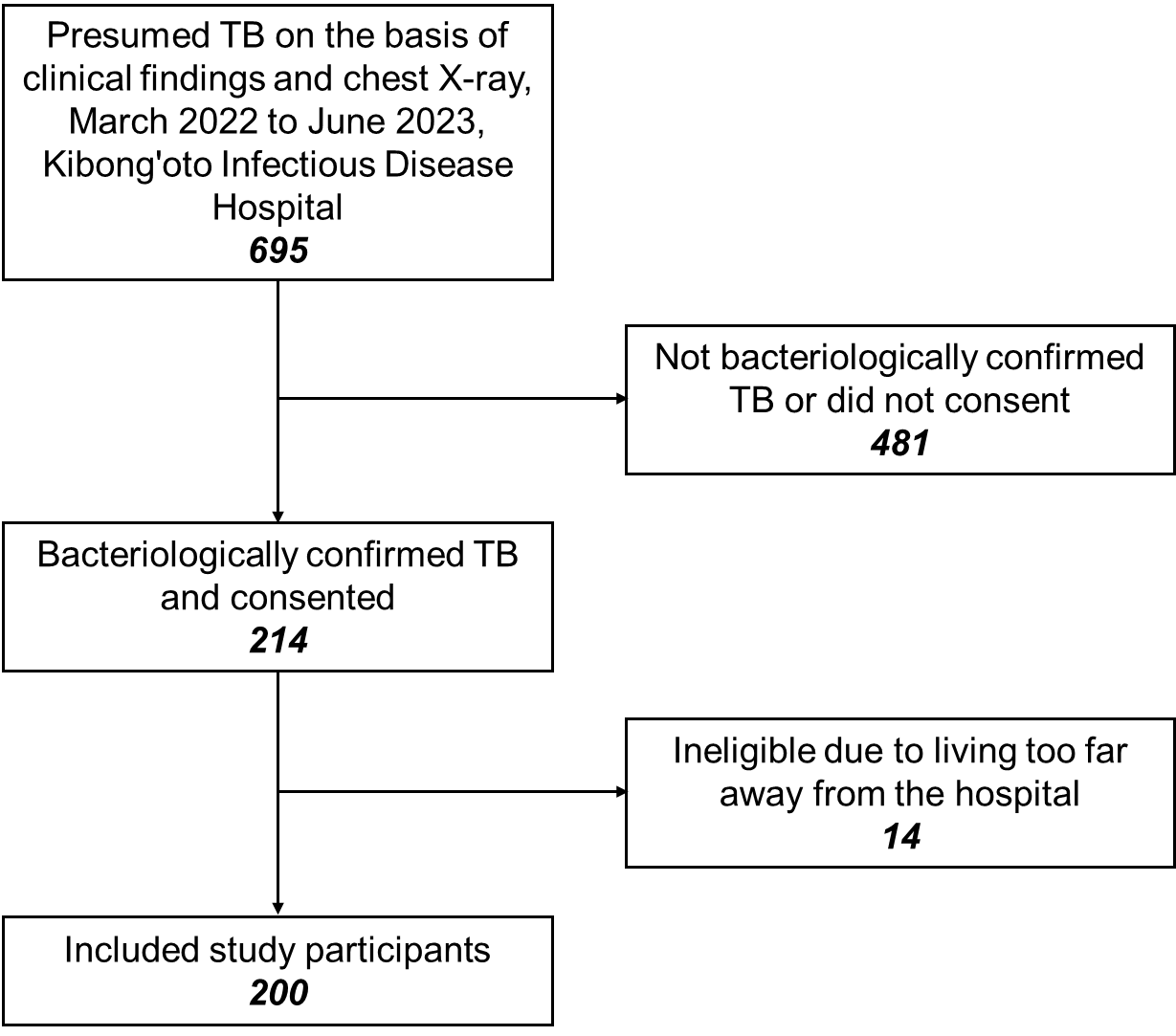


## Figure S2. Missed dose gaps among those with sporadic missed doses (N=110).

Scatter plot of the median (A) and maximum (B) length of gaps for an individual against the number of gaps of any length for an individual, using the sensitivity analysis missed dose variable.

## Figure S3. Differences in pillbox data and monthly pill counts

This graph illustrates the probability distribution of the proportion of doses taken for the two pillbox measures- i.e. the main missed dose variable (blue) and sensitivity missed dose variable (red)- and monthly pill count data (green).

## Figure S4. Patterns in timings of doses taken, for selected participants.

Participants selected on the basis of displaying different temporal patterns. The blue line charts the first time each day that the pillbox was opened. The red dots indicate additional openings within that day. Panels A-C) are individuals with ≤10% of doses missed and D-G) are individuals with >10% of doses missed.


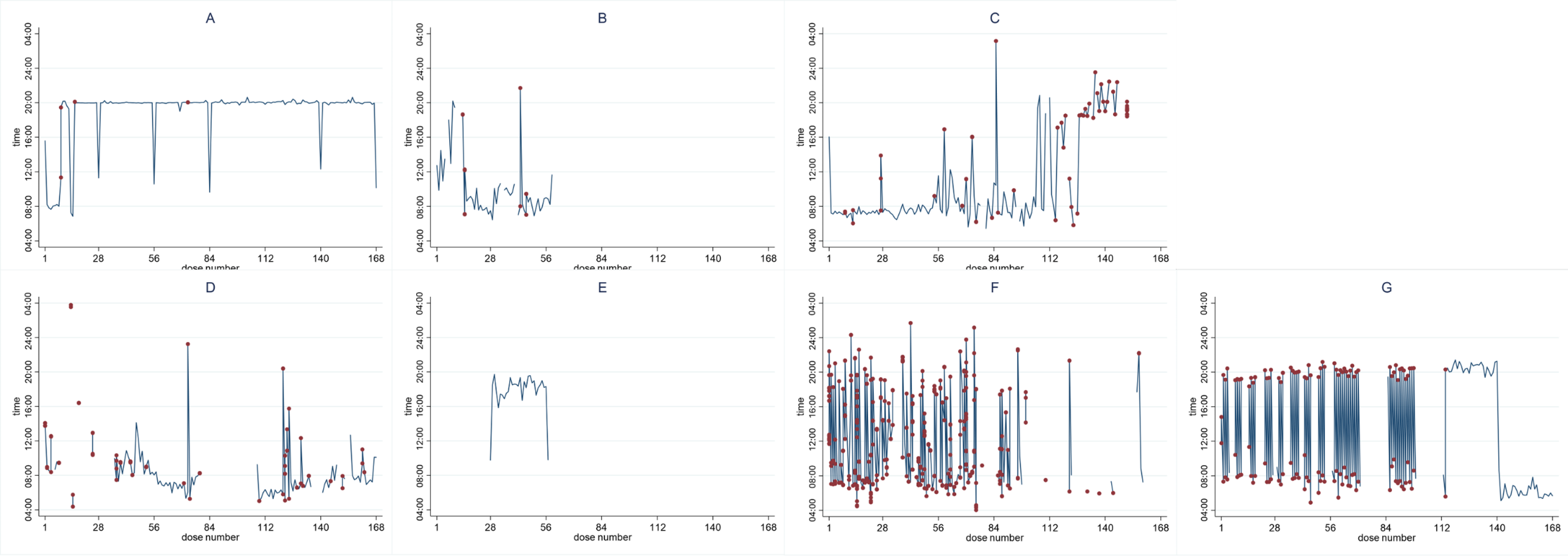


Figure S5. Lasagna plots of the reasons for missed doses

Lasagna plots of the reason for missed dose periods for each participant: (A) any reason, (B) forgot, (C) felt well, (D) inconvenient, (E) pocket dose, (F) side effect/unwell, (G) unable/lack of support. Each row in the figure represents a participant, ordered by percentage of doses taken. This was calculated as a percentage of total doses during treatment period (e.g., 168 doses) accounting for missing information and then grouped: <80%, 80-89%, 90-99%, and 100%. Rows are coloured by dose taken groups, as per the legend. Blue indicates doses taken. Grey indicates when pillbox data was missing or unreliable (e.g. box failure or lost box). Dark red indicates the specific reason. Light red indicates another reason, not the specified reason.

A. B. C.

 
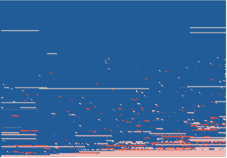

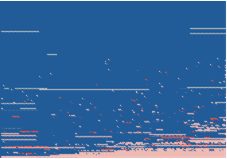

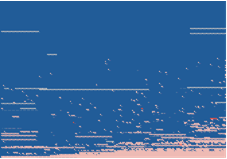


D. E. F.


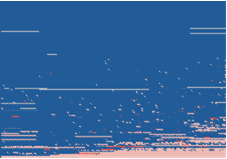

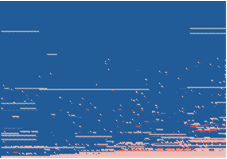

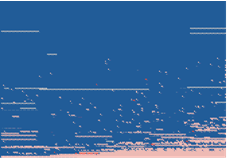


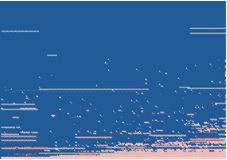
 G.
